# Supplementary material for: Under-5 Mortality and Its Associated Factors in Northern Nigeria: Evidence from 22,455 Singleton Live Births (2013–2018)
Source: Int J Environ Res Public Health. 2021 Sep 20;18(18):9899. doi: 10.3390/ijerph18189899 (PMC8469194; doi:10.3390/ijerph18189899)
Supplement: Supplementary file 1 [file ijerph-18-09899-s001.zip › ijerph-1362468-supplementary.pdf]

International Journal of *Environmental Research and Public Health*

MDPI

Table S1. Hierarchical multivariable model ¥.

**Variable**	**Model 0**	**Model 1**	**Model 2**	**Model 3**	**Model 4**
Community level factor	OR(95%CI)	aOR(95%CI)	aOR(95%CI)	aOR(95%CI)	aOR(95%CI)
**Residence type**					
Urban	Ref	Ref	Ref		
Rural	1.35(1.14—1.60)	1.32 (1.13—1.53)^	1.02(0.88—1.20)	—	—
**Geopolitical zone**					
North Central	Ref	Ref	Ref	Ref	Ref
North East	1.30(1.08—1.55)	1.25 (1.05—1.48)^	1.08(0.90—1.30)	1.05 (0.87—1.26)	1.07 (0.89—1.29)
North West	1.71(1.45—2.01)	1.66 (1.42—1.94^)	1.41(1.18—1.68)^	1.34 (1.13—1.59)^	1.38(1.16—1.65)^
Socioeconomic level factor					
**Household wealth index**					
Rich	Ref		Ref	Ref	Ref
Middle	1.73(1.22—2.45)	—	1.41(0.99—1.98)	1.38 (0.98—2.02)	1.44(1.01—2.07)^
Poor	2.37(1.71—3.30)	—	1.67(1.18—2.36)^	1.65(1.14—2.39)^	1.68(1.17—2.42)^
**Mother's education**					
Secondary or higher	Ref		Ref	Ref	Ref
Primary	1.52(1.24—1.86)	—	1.21(0.95—1.55)	1.28 (1.02—1.61)^	1.29(1.03—1.62)^
No education	1.92(1.61—2.30)	—	1.29(1.01—1.70)^	1.38 (1.10—1.73)^	1.35(1.07—1.70)^
**Mother's literacy level**					
Able to read	Ref		Ref		

Cannot read	1.71(1.47—1.99)	—	1.06(0.86—1.30)	—	—
**Father's education**					
Secondary or higher	Ref		Ref	Ref	Ref
Primary	1.48(1.23—1.78)	—	1.22(1.01—1.48)^	1.21 (1.00—1.47)	1.21(1.01—1.46)^
No education	1.73(1.51—1.99)	—	1.37(1.17—1.61)^	1.31 (1.12—1.53)^	1.32(1.13—1.54)^
**Father's occupation**					
Not working	Ref		Ref	Ref	Ref
Non-agricultural work	1.37(0.99—1.87)	—	1.62(1.20—2.21)^	1.57 (1.15—2.15)^	1.58(1.17—2.15)^
Agricultural work	1.51(1.11—2.06)	—	1.41(1.04—1.91)^	1.42 (1.04—1.94)^	1.44(1.06—1.96)^
**Number of women in household**					
One woman	Ref		Ref		
At least 2 women	1.17(1.04—1.31)	—	1.08(0.96—1.21)	—	—
Individual related factor					
**Mother's age**					
< 20	1.60(1.24—2.05)	—	—	1.17 (0.87—1.57)	—
20—29	1.08(0.97—1.21)	—	—	1.00 (0.87—1.15)	—
30—39	Ref			Ref	
40—49	1.21(1.02—1.42)	—	—	1.15 (0.97—1.36)	—
**Mother's body mass index kg/m² (MBMI)**					
Normal (18.5≤MBMI≥24.9)	Ref			Ref	
Underweight (MBMI < 18.5)	1.06(0.94—1.19)	—	—	1.04 (0.93—1.17)	—
Overweight (25≤MBMI≥29.9)	1.07(0.88—1.29)	—	—	1.21 (0.99—1.47)	—
Obese (MBMI≥30)	0.85(0.53—1.37)	—	—	1.35 (0.83—2.19)	—
**Wanted pregnancy**					
Wanted then	Ref			Ref	
Wanted later	0.83(0.64—1.09)	—	—	0.93 (0.71—1.23)	—
Wanted no more	0.71(0.44—1.14)	—	—	0.771 (0.48—1.25)	—
**Mother's perceived baby size**					

Average or larger	Ref			Ref	Ref
Small or very small	1.44(1.22—1.69)	—	—	1.37 (1.19—1.58)^	1.34(1.16—1.55)^
**Sex**					
Female	Ref			Ref	
Male	1.07(0.95—1.22)	—	—	1.08 (0.94—1.23)	—
**Birth order/ birth interval**					
First	1.63(1.39—1.91)	—	—	1.57 (1.33—1.85)^	1.61(1.38—1.88)^
2nd or 3rd rank, interval ≤ 2 yr	1.82(1.45—2.30)	—	—	1.75 (1.39—2.19)^	1.79(1.43—2.23)^
2nd or 3rd rank, interval > 2 yr	Ref			Ref	Ref
4th or higher rank, interval ≤ 2 yr	1.17(1.01—1.36)	—	—	1.03 (0.86—1.24)	1.07 (0.92—1.24)
4th or higher rank, interval > 2 yr	1.92(1.63—2.27)	—	—	1.65 (1.35—2.01)^	1.68(1.42—1.98)^
Health related factor					
**Contraceptive use**					
Yes	Ref				Ref
No	1.87(1.53—2.29)	—	—	—	1.43(1.14—1.78)^
**Place of birth**					
Home	1.36(1.17—1.58)	—	—	—	0.85 (0.60—1.20)
Health facility	Ref				Ref
**Mode of delivery**					
Non-caesarean	Ref				Ref
Caesarean	1.58(0.93—2.69)	—	—	—	2.52(1.47—4.32)^
**Delivery assistance**					
Health professional	Ref				Ref
Non-health professional	1.40(1.21—1.61)	—	—	—	1.06 (0.76—1.48)

Notes: ^, significant variable (s) added to the next model; Model 0 – Unadjusted for all the independent variables; Model 1- Community level factors (residence type & region); Model 2 - significant variable(s) in Model 1 added to socioeconomic variables (household wealth status, maternal education, paternal education, mother literacy level, father's occupation, number of women in the household); Model 3 – significant variable(s) in Model 2 added to individual level factor (mother's age, MBMI, wanted pregnancy at the time pregnant, perceived baby size by their mothers, child sex, & birth order/interval); Model 4 – significant variables in Model 3 added to health related factor (contraceptive use, delivery assistant, mode of delivery & place of delivery). yr, years; aOR (95%CI): adjusted Odds ratio with corresponding 95% confidence interval, OR: unadjusted Odds ratio; Ref, reference category; §, Caesarean section is a combination of elective and emergency procedures; ¥, 1,113 missing values were excluded from the analyses. .
